# A Suture-specific Photo Score for Metopic Synostosis

**DOI:** 10.1097/SCS.0000000000009773

**Published:** 2023-10-10

**Authors:** Linda Gaillard, ERN CRANIO–Craniosynostosis Workgroup

**Affiliations:** Department of Plastic and Reconstructive and Hand Surgery, Dutch Craniofacial Centre, Erasmus MC, Sophia Children’s Hospital, University Medical Centre Rotterdam, Rotterdam, the Netherlands

**Keywords:** Esthetic, metopic synostosis, phenotype, photo score, trigonocephaly

## Abstract

Head shape assessments in children with metopic synostosis are a relevant outcome measure in addition to functional measures, such as neurocognitive outcomes, behavioral outcomes, and visual functioning outcomes. However, consensus on head shape assessments in children with metopic synostosis is lacking. The aim of this study is to develop a reproducible and reliable suture-specific photo score that can be used for cross-center comparison of phenotypical severity of metopic synostosis and evaluation of esthetic outcome of treatment later in childhood. We conducted a retrospective study among nonsyndromic metopic synostosis patients aged <18 years. Preoperative and postoperative photosets of patients with metopic synostosis from 6 expert centers were included. The photo score was discussed in the group of expert craniofacial plastic surgeons and pediatric neurosurgeons. Interrater reliability was determined with modified weighted Fleiss’ kappa and intraclass correlation coefficients. Correlation between individual photo score items with overall phenotype was assessed using Spearman correlation analyses. The metopic synostosis photo score contained the following items: “wedging of the forehead”, “hypotelorism”, “temporal hollowing”, “biparietal widening,”and an assessment of “overall phenotype”. Items were scored on a 4-point ordinal scale ranging from normal to severe. We found moderate interrater reliability for all items, but substantial agreement for the summed scores. Correlation with overall phenotype was lowest for biparietal widening. To conclude, although agreement on individual photo score items was suboptimal, the agreement on the summed score was substantial, which indicates there is consensus on the overall severity of the metopic synostosis phenotype.

The phenotypical severity of metopic synostosis varies considerably depending on the timing of metopic suture fusion during fetal development.^1^ The phenotype can range from a clinically insignificant metopic ridge to a true trigonocephaly phenotype with a wedge-shaped forehead, hypotelorism, temporal hollowing, and an overall triangular skull shape. To assess the severity of metopic synostosis, several measures have been described previously. These include anthropometric measures on CT scans such as the interfrontal angle (angle between bilateral pterion to nasion lines), the amount of frontal stenosis (interparietal distance / intercoronal distance), and the recently developed quantitative shape severity score, as well as measures acquired through 3D imaging systems.^2–9^ However, CT scans require radiation exposure and are consequently rarely repeated postoperatively, and consensus on what constitutes the best anthropometric measure is lacking.^8^ 3D imaging systems are not easily accessible for all health care providers as they require expensive equipment and expertise in analyses of data obtained. A simple, reliable 2D photo score would therefore be valuable to assess the phenotypical severity of the metopic synostosis. Previous studies that have compared esthetic outcome or phenotypical severity often used the Whitaker score,^10^ which is a general score for assessing esthetic outcomes after craniosynostosis surgery.^11–13^ The main disadvantage of this score is that it only assesses general esthetic outcomes and the need for reintervention after surgery rather than the severity of phenotypical characteristics.

Adequately classifying the severity of the trigonocephaly phenotype is especially important given the ongoing debate regarding the need for surgical intervention. Historically, the standard of care has been cranial vault surgery with the aim of reducing potential mechanical brain restriction and correcting the skull contours. However, the functional indication for surgical intervention in metopic synostosis is unclear, and to what extent surgical intervention improves (long-term) esthetic outcomes compared with conservative management is unknown. To assess the severity of characteristic trigonocephaly features in metopic synostosis, we aimed to develop a suture-specific photo score and to assess its reliability in an expert group of craniofacial plastic surgeons and neurosurgeons. This photo score can potentially contribute to accurately comparing phenotypical severity at presentation and esthetic outcome from different treatment strategies across expert centers in the future.

## METHODS

### Study Design and Subjects

We conducted a retrospective study among nonsyndromic metopic synostosis patients. Six expert centers supplied photographs for photo score assessments: Birmingham Children’s Hospital (Birmingham, UK), Charité-Universitätsmedizin Berlin (Berlin, Germany), Erasmus Medical Center, Sophia Children’s Hospital (Rotterdam, The Netherlands), Fondazione IRCCS Istituto Neurologico Carlo Besta (Milan, Italy), Hôpital Necker-Enfants-Malades (Paris, France), Hospital 12 de Octubre (Madrid, Spain). Photosets of patients < 18 years, available in 4directions (anterior-posterior view, both lateral views, bird’s eye view) were included. We included preoperative and postoperative photosets that were mixed randomly for scoring. A panel of expert craniofacial plastic surgeons and neurosurgeons who are members of the craniosynostosis workgroup of the European Reference Network CRANIO (ERN CRANIO) evaluated photosets independently. ERN CRANIO is a network of health care providers across the European Union and European Economic Area, focused on complex craniofacial anomalies, including craniosynostosis, and/or rare ear, nose, and throat disorders.^14^ To qualify as a member of the ERN CRANIO–craniosynostosis workgroup, a minimum of 20 intracranial surgeries on nonsyndromic unisutural craniosynostosis patients should be performed yearly, a multidisciplinary team is required, and national acknowledgment by the Health Authority is mandatory.

### Photo Score Development and Assessments

The photo score development process was described in detail for a previous sagittal synostosis photo score in Gaillard et al 2023 (submitted). In brief, 2 meetings with experts were organized to discuss which items should be included in the photo score and to do a pilot study. After obtaining consensus on the photo score, a panel of expert craniofacial plastic surgeons and neurosurgeons used the developed photo score to evaluate new photosets independently to assess the reliability of the photo score. All participants received identical instructions and were shown example photos of patients with features considered “severe” for each item. All items in the photo score were scored according to the same 4-point scale, which ranged from normal to severe. Participants scored 2 initial practice photosets, of which results were discussed immediately to clarify any remaining uncertainties regarding the score before rating 42 study photosets. Participants were shown photosets through Microsoft Teams software, and photosets were scored using Mentimeter.

### Statistical Analysis

Statistical analyses were performed using R version 4.1.1 (2021-08-10). The panel’s interrater reliability was assessed using the wlin.conc() function in the package ‘raters’ in R. We obtained a modified version of Fleiss’ kappa with linear weights and calculated 95% confidence intervals (CI) using the percentile bootstrap method with 10, 000 iterations.^15^ Second, pairwise weighted kappa analyses were performed for each combination of surgeons with the kappa2() function with equal weights of the package ‘irr’. In addition, the sum score of individual photo score items was calculated. The intraclass correlation coefficient (ICC) estimates for consistency and agreement for the summed scores were obtained based on single rating, 2-way random effects models. Kappa and ICC values were interpreted according to the Landis and Koch scale.^16^ Finally, we investigated the relation between each individual photo score item with “overall phenotype severity” assessments using scatter plots.

In a secondary analysis, we assessed if removing the individual item with the lowest correlation with overall phenotype affected the interrater reliability of the summed score by calculating the ICC measures as described above without the low correlation item. In addition, we obtained the interrater reliability within a single center, if multiple surgeons from a single center participated in the study to compare single-center results to our full panel of plastic surgeons and neurosurgeons using the wlin.conc() function in the package “raters” in R. Finally, the interrater reliability for high-quality photosets was calculated. Photosets were considered high quality when they met the following criteria: no hair obstructing view of craniofacial features, optimal angle of photo, and optimal lighting.

## Results

### Photo Score Development

**FIGURE 1:**
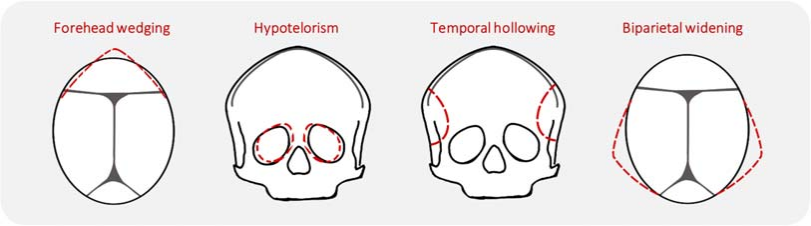
Schematic figure with characteristic trigonocephaly features indicated in the red dotted lines.

**FIGURE 2:**
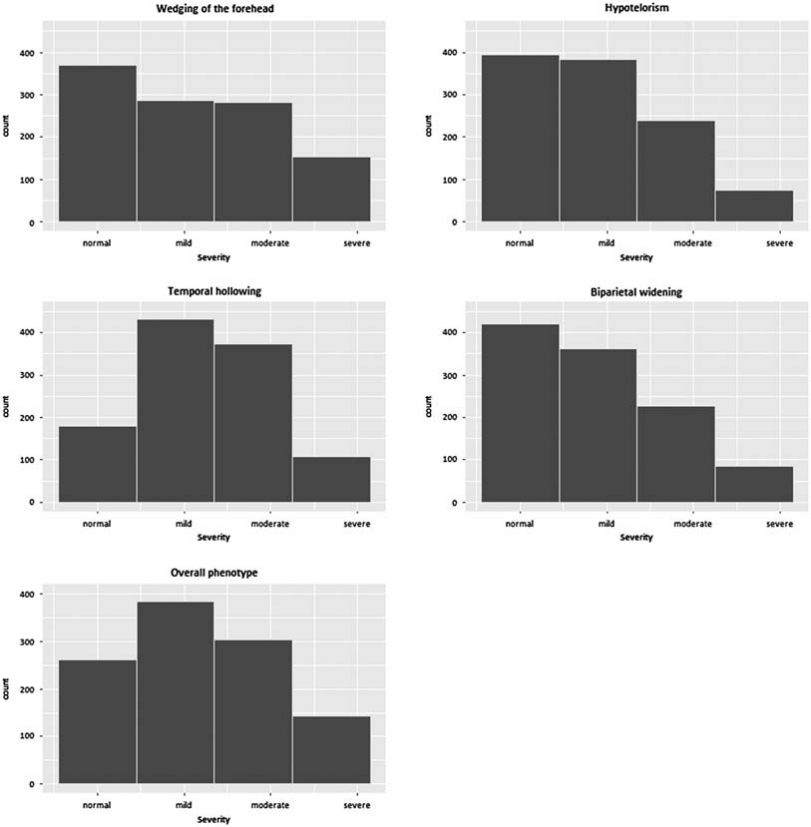
Total scores for each category.

We obtained consensus on a photo score that contains 4 characteristic trigonocephaly features as well as an assessment of the severity of the ‘Overall phenotype’ (Supplemental Table 1, Supplemental Digital Content 1, http://links.lww.com/SCS/F551, Fig. 1). We used an ordinal 4-point scale with “0” indicating a normal feature, “1” indicating a mild deformity, “2” indicating moderate deformity, and “3” indicating a severe deformity. How often each category was scored is shown in Fig. 2.

### Photo Score Assessments

#### Interrater Reliability

**FIGURE 3:**
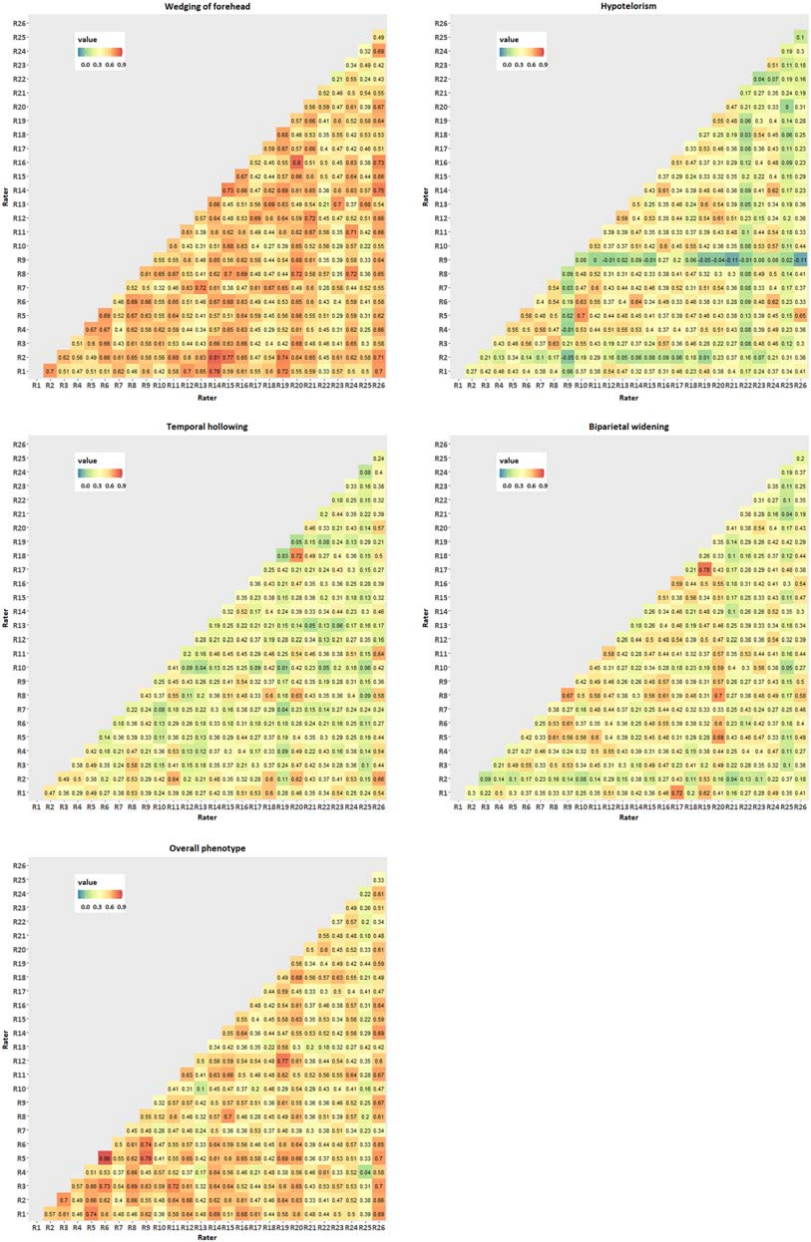
Pairwise kappa.

Forty-two photosets were scored by 26 surgeons. Interrater reliability was moderate for all items and was highest for the “wedging of the forehead” and “overall phenotype” (Supplemental Table 2, Supplemental Digital Content 1, http://links.lww.com/SCS/F551). Interrater reliability was higher when using the summed score with an agreement ICC estimate of 0.67 (CI 0.57, 0.77), indicating substantial agreement. Pairwise kappa analyses for each set of raters indicate substantial variation with agreement strength ranging from poor agreement to almost perfect agreement. The variation in interrater reliability was especially large for “hypotelorism” (κ min=−0.11, κ max=0.70), “temporal hollowing” (κ min=0.01, κ max=0.72), and “biparietal widening” (κ min=0.04, κ max=0.78) (Fig. 3). Interrater reliability for 10 high-quality photographs was similar to interrater reliability of all photosets (Supplemental Table 3, Supplemental Digital Content 1, http://links.lww.com/SCS/F551).

#### Relation Between Subitems and Overall Phenotype Assessment

**FIGURE 4:**
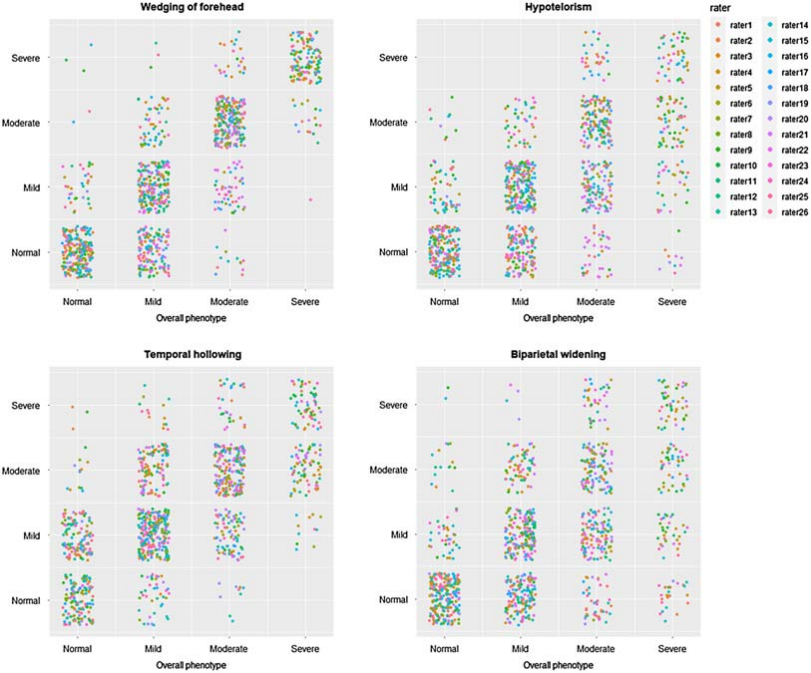
Correlation between individual photo score items and overall phenotype.

In line with the variation in pairwise kappa analyses, the correlation between individual items and the “overall phenotype” assessments also varied considerably between raters. “Biparietal widening” had the lowest correlation with overall phenotype. This is shown in Fig. 4, which shows scatter plots indicating the relation between individual items and the overall severity. The figure indicates a strong relation between “Wedging of the forehead” and “Overall phenotype,” and the weakest relation between “Biparietal widening’ and “Overall phenotype”. The interrater reliability for the summed score without the “Biparietal widening” item was substantial [ICC agreement: 0.68 (95% CI 0.58, 0.78), ICC consistency 0.74 (95% CI 0.65, 0.82)].

#### Single Center Interrater Reliability

In 5 centers, multiple surgeons from the same center scored the photo sets independently (in 1 center 4 surgeons participated and in 4 centers 2 surgeons participated). Interrater reliability was similar or higher to the total group interrater reliability for most items in 4 centers. In these centers, the minimum agreement type ICC for the summed score was 0.69 (95% CI: 0.49, 0.82) and maximum agreement type ICC was 0.81 (95% CI 0.67, 0.89). For the fifth center, the agreement type ICC of the summed score was lower [0.29 (95% CI: −0.07, 0.59)] than the total group ICC. Modified Fleiss’ kappa statistics for individual items showed similar variation to pairwise kappa analyses and ranged from poor agreement to substantial agreement.

## DISCUSSION

We aimed to develop a photo score that can be used by experts to assess the severity of characteristic trigonocephaly features in metopic synostosis patients. Our study indicates disagreement between experts on the interpretation of specific photo score items despite consensus on the photo score itself.

Several studies have investigated photo scores to assess the esthetic outcome of metopic synostosis surgery.^11–13,17–21^ However, many scores are not applicable preoperatively as they assess the effect of surgical intervention on esthetic outcome and the need for reintervention or only focus on specific features of the trigonocephaly phenotype.^11,12,18,13,20,22^ In addition, 2 studies investigated if parents were satisfied with results from either conservative management or surgical intervention.^19,21^ To our knowledge, only 1 previous study used a 2D photo score that assesses true phenotypical severity of the metopic synostosis phenotype.^17^ A junior plastic surgeon and medical student scored photographs on the shape of the forehead, hypotelorism, and temporal depression on a 3-point scale. The authors report substantial to almost perfect interrater reliability on the Landis and Koch scale for each item (κ statistic ranging from 0.65 (temporal depression) to 0.89 (shape of forehead). Similarly, we found substantial agreement for the summed score. However, both the total group interrater reliability, as well as the interrater reliability of centers in which multiple surgeons participated in our study, is lower for similar individual photo score items. The difference in interrater reliability, even in a single center setting, may be explained by different rating scales. In the current study, photographs were scored on a 4-point scale. Although a 4 -4-point scale is more informative to communicate the direction of the severity of the phenotype, the elimination of the neutral middle choice likely negatively impacts the interrater reliability measures. Although some photosets were of suboptimal quality, the moderate interrater reliability reflects a true difference in the interpretation of the photo score items by experts, as a subanalysis of high-quality photos did not improve interrater reliability. These findings highlight the subjective nature of esthetic assessments, even if assessments are made by expert raters. Although a subanalysis of high-quality photosets did not improve interrater reliability, consistent, uniform photographs may facilitate easier and more uniform scoring, and standardized photograph-taking should ideally be implemented across participating centers. In addition, an educational program for participants could be implemented using data and examples from our current study.

Although all individual items had moderate interrater reliability. “Biparietal widening” correlated least with the overall phenotype. Therefore, we recommend the final score includes the following individual items: “Wedging of the forehead”, “Hypotelorism” and “Temporal hollowing”. Biparietal widening likely correlated less with overall phenotype as it can be difficult to assess on photographs of patients with hair.

To accurately compare different treatment strategies between expert centers, a consistent rating and analysis of both preoperative phenotypical severity and postoperative esthetic outcome is needed as the severity of the phenotype at presentation likely predicts esthetic outcome after treatment. Recent studies have shown the potential of 3D photogrammetry as a radiation-free alternative to CT in assessing the severity of metopic synostosis and for morphometric follow-up.^3,23–26^ 3D shape analyses will likely provide more information on phenotypical severity and the effect of treatment on head shape, especially if 3D photogrammetry can be analyzed objectively and consistently through the use of artificial intelligence. Combining objective 3D photogrammetry shape analyses with the current photo score would allow us to validate and improve the 2D photo score further. In contrast to 3D photogrammetry, which can be difficult to access due to the required expensive equipment and expertise in analysis, 2D photographs are highly accessible to all clinicians. As the 2D photographs are easily obtained by clinicians, the 2D photo score could be widely used throughout expert centers and allow for benchmarking of esthetic results after different treatment strategies. Combined with studies on neurocognitive outcomes, behavioral outcomes, visual functioning, as well as the quality of life of patients and their parents, a widely-used reliable photo score will contribute to developing an optimized treatment protocol tailored to the severity of metopic synostosis if necessary.

## Conclusion

Agreement on the overall phenotype determined by the summed score of individual photo score items was substantial despite moderate agreement on individual photo score items, indicating that despite disagreement between experts on the interpretation of individual photo score items, experts agree on the overall phenotypical severity of metopic synostosis.

## Supplementary Material

SUPPLEMENTARY MATERIAL
