# Supplementary material for: A Suture-specific Photo Score for Metopic Synostosis
Source: J Craniofac Surg. 2023 Oct 10;35(1):75–9. doi: 10.1097/SCS.0000000000009773 (PMC10749672; doi:10.1097/SCS.0000000000009773)
Supplement: SUPPLEMENTARY MATERIAL [file scs-35-075-s001.docx]

| **Metopic synostosis photo score** | **View** |
| --- | --- |
| **Wedging of the forehead**  A pointed appearance of the forehead, the phenotype is considered more severe if the midline of the forehead is more pronounced and with a more acute angle. | Bird’s eye |
| **Hypotelorism**  Abnormally small interocular distance. | Anterior-posterior |
| **Temporal hollowing**  Abnormal temporal hollowing/pinching above the supraorbital bar on the lateral sides of the forehead. | Anterior-posterior |
| **Biparietal widening**  Abnormal biparietal widening, increased widening is considered a more severe phenotype. | Bird’s eye |
| **Overall phenotype**  Severity of the overall phenotype | All |

| **Scale** | **Normal** | **Mild** | **Moderate** | **Severe** |
| --- | --- | --- | --- | --- |

**Supplemental Table 1. Metopic synostosis photo score.** The minimum score was 0 points (‘normal’ phenotype) and the maximum total score, excluding “overall phenotype”, was 12 points (with all features rated as ‘severe’). All items were scored on a 4-point scale.

**Supplemental Table 2. Interrater reliability**

| **Photo score item** | **Modified Fleiss’ kappa statistic** | **95% bootstrap CI** |
| --- | --- | --- |
| Wedging of the forehead | 0.57 | 0.53 ; 0.63 |
| Hypotelorism | 0.46 | 0.40 ; 0.52 |
| Temporal hollowing | 0.47 | 0.43 ; 0.50 |
| Biparietal widening | 0.46 | 0.41 ; 0.52 |
| Overall phenotype | 0.56 | 0.53 ; 0.60 |

**Supplemental Table 2A.** Interrater reliability of each item in the photo score. All items have a moderate agreement strength on Landis and Koch scale.

| **Intraclass correlation coefficient type** | **ICC** | **95% CI** |
| --- | --- | --- |
| Agreement | 0.67 | 0.57 ; 0.77 |
| Consistency | 0.74 | 0.66 ; 0.83 |

**Supplemental Table 2B.** Intraclass correlation coefficient for sum scores (excluding *‘Overall phenotype’* ). Both are considered substantial agreement strength on Landis and Koch scale.

Abbreviations: ICC = intraclass correlation coefficient, CI= confidence interval.

**Supplemental Table 3 Interrater reliability for high quality photos**

| **Photo score item** | **Modified Fleiss’ kappa statistic** | **95% bootstrap CI** |
| --- | --- | --- |
| Wedging of the forehead | 0.58 | 0.49 ; 0.67 |
| Hypotelorism | 0.37 | 0.30 ; 0.43 |
| Temporal hollowing | 0.44 | 0.37 ; 0.52 |
| Biparietal widening | 0.46 | 0.37 ; 0.55 |
| Overall phenotype | 0.53 | 0.48 ; 0.59 |

**Supplemental Table 3A.** Interrater reliability of each item in the photo score for ten high quality photo sets. All items have a moderate agreement strength on Landis and Koch scale.

| **Intraclass correlation coefficient type** | **ICC** | **95% CI** |
| --- | --- | --- |
| Agreement | 0.61 | 0.40 ; 0.84 |
| Consistency | 0.74 | 0.57 ; 0.91 |

**Supplemental Table 3B.** Intraclass correlation coefficient for sum scores of ten high quality photo sets (excluding *‘Overall phenotype’* ). Both are considered substantial agreement strength on Landis and Koch scale.

Abbreviations: ICC = intraclass correlation coefficient, CI= confidence interval.
